# Isolation of Rare Tumor Cells from Blood Cells with Buoyant Immuno-Microbubbles

**DOI:** 10.1371/journal.pone.0058017

**Published:** 2013-03-13

**Authors:** Guixin Shi, Wenjin Cui, Michael Benchimol, Yu-Tsueng Liu, Robert F. Mattrey, Rajesh Mukthavaram, Santosh Kesari, Sadik C. Esener, Dmitri Simberg

**Affiliations:** 1 Moores Cancer Center, University of California San Diego, La Jolla, California, United States of America; 2 Department of Radiology, University of California San Diego, San Diego, California, United States of America; 3 Department of Electrical and Computer Engineering, University of California San Diego, La Jolla, California, United States of America; 4 Neuro-Oncology Program, Moores Cancer Center, University of California San Diego, La Jolla, California, United States of America; 5 Department of Neurosciences, University of California San Diego, La Jolla, California, United States of America; 6 Department of NanoEngineering, University of California San Diego, La Jolla, California, United States of America; 7 Solid Tumor Therapeutics Program, Moores Cancer Center, University of California San Diego, La Jolla, California, United States of America; Northwestern University Feinberg School of Medicine, United States of America

## Abstract

Circulating tumor cells (CTCs) are exfoliated at various stages of cancer, and could provide invaluable information for the diagnosis and prognosis of cancers. There is an urgent need for the development of cost-efficient and scalable technologies for rare CTC enrichment from blood. Here we report a novel method for isolation of rare tumor cells from excess of blood cells using gas-filled buoyant immuno-microbubbles (MBs). MBs were prepared by emulsification of perfluorocarbon gas in phospholipids and decorated with anti-epithelial cell adhesion molecule (EpCAM) antibody. EpCAM-targeted MBs efficiently (85%) and rapidly (within 15 minutes) bound to various epithelial tumor cells suspended in cell medium. EpCAM-targeted MBs efficiently (88%) isolated frequent tumor cells that were spiked at 100,000 cells/ml into plasma-depleted blood. Anti-EpCAM MBs efficiently (>77%) isolated rare mouse breast 4T1, human prostate PC-3 and pancreatic cancer BxPC-3 cells spiked into 1, 3 and 7 ml (respectively) of plasma-depleted blood. Using EpCAM targeted MBs CTCs from metastatic cancer patients were isolated, suggesting that this technique could be developed into a valuable clinical tool for isolation, enumeration and analysis of rare cells.

## Introduction

As cancer progresses, malignant cells are shed into the blood [1], [2], [3]. Circulating tumor cells (CTCs) could provide invaluable information for the monitoring of tumor progression and recurrence in cancer patients [1], [2], [3]. The successful identification and isolation of CTCs is a major challenge akin to finding a needle in a haystack: there are only a few CTCs per million of blood cells circulating throughout the body.

At present, several assays for CTC isolation and analysis are on the market or in clinical development. The most common strategy for isolating CTCs from blood is based on the use of immunomagnetic beads coated with anti-epithelial EpCAM [4], [5], [6], [7], [8], the most commonly used marker for detecting circulating tumor cells [7], [9]. An immunomagnetic bead-based CellSearch Assay (Veridex) has received U.S. Food and Drug Administration approval for the detection of epithelial CTCs in metastatic cancer patients. At present, this assay is the gold standard for CTC isolation. The capturing efficiency of rare tumor cells with magnetic beads ranges between 60–90% [10], [11]. The most significant limitations of the assay are its relatively long processing time, non-specific carryover and contamination with leukocytes [8], [12], [13], [14]. Recently, the field of CTC isolation witnessed a surge of technologies, including microfluidics and filtration. These state-of-the-art technologies allow to isolate, count and even to manipulate single CTCs [15], [16], [17], [18]. At the same time, there is a continuing interest in development and testing of cost-efficient, scalable and simple technologies for CTC isolation.

Perfluorocarbon gas-filled microbubbles (MBs) are clinically approved for injection as ultrasound contrast agents [19], [20]. A typical microbubble consists of a gas interior coated by a soft shell, which could consist of either a lipid monolayer or protein (albumin). Perfluorocarbon gas maintains the stability of MBs in the aqueous phase and confers buoyancy [19]. Recently, we demonstrated that anti-fluorescein antibody-coated buoyant MBs efficiently bound and separated fluorescein-labeled erythrocytes in mouse blood [21]. Here we set out to test whether EpCAM-targeted MBs are capable of sensitive and specific isolation of rare tumor cells from mouse and human blood. Our data suggest that MBs efficiently and specifically isolate tumor cells from plasma-depleted blood. We demonstrate that buoyancy-based separation of tumor cells from complex cell mixtures is feasible and could become a promising strategy to immune marker-based fractionation and isolation of rare cells.

## Materials and Methods

### 1. Ethics statement

Collection of healthy blood from anonymous volunteers was approved by the UC San Diego Institutional Review Board (protocol 081077XT). Collection and usage of human specimens from consenting patients was approved by the UC San Diego Institutional Review Board (protocol 100936). All the participants had to sign approved IRB approved consent form prior to blood collection. All animal studies were conducted under UCSD IACUC protocol (protocol S07388).

### 2. Reagents

1,2-distearoyl-sn-glycero-3-phosphocholine (DSPC) was purchased from Avanti Polar Lipids (Alabaster, AL, USA), 2-distearoyl-sn-glycero-3-phospho-ethanolamine-N-[maleimide(polyethylene glycol)-3400] (DSPE-PEG3400-Malemide) and maleimide-polyethylene glycol 3400-succinimidyl valerate (Mal-PEG-SVA) were purchased from Laysan Bio, Inc. (Arab, AL, USA), polyoxyethylene (40) stearate was purchased from Sigma. All lipids were stored as chloroform solution under argon at −20°C. Traut's reagent (2−Iminothiolane) was purchased from Thermo Fisher Scientific (Rockford, IL, USA). The reagent was dissolved in double-distilled water at 5 mg/ml and stored in aliquots at −20°C. Ellman's reagent (5,5'-dithiobis-(2-nitrobenzoic acid), or DTNB) was purchased from Thermo Fisher Scientific and stored as a dry powder at −4°C prior to use. Nuclear stain Hoechst 33342 trihydrochloride trihydrate (Invitrogen, Carlsbad, CA, USA) was stored frozen as a 1 mg/ml solution in PBS. AffiniPure Rabbit Anti-Mouse IgG, Fc Fragment Specific and ChromPure Rabbit IgG, whole molecule was purchased from Jackson ImmunoResearch (West Grove, PA, USA). Mouse anti-human CD326 (EpCAM) antibodies was purchased from Bio Legend (San Diego, CA, USA), as was purified rat anti-mouse CD326 antibody. Alexa Fluor 488 mouse anti-pan Cytokeratin antibody (clone AE1/AE3) was purchased from eBioscience (San Diego, CA, USA). All antibodies were stored at 4°C prior to use. Zeba Spin Desalting Columns were purchased from Thermo Fisher Scientific.

### 3. MB and immunomagnetic bead preparation

MBs were prepared from a mixture of DSPC/PEG40 stearate/DSPE-PEG3400-maleimide as described [21]. Briefly, lipids in chloroform were mixed at 10∶1∶1 molar ratio in a 2 ml borosilicate glass vial (100–300 nmoles total lipid) and dried under an argon stream to form a thin lipid film. The film was rehydrated in 1 ml phosphate-buffered saline (PBS, pH 7.4) at room temperature for 5 minutes. The lipid was further dispersed under gas perfluorohexane (Alfa Aesar, Ward Hill, MA, USA) atmosphere by sonication (30-second cycle, 3–5 cycles total) using a MISONIX XL-2000 probe sonicator at power setting ‘1’ Excess phospholipid membrane fragments and small MBs were removed by centrifugation at 50 g for 1 minute, repeated three times. MBs were resuspended in PBS at a concentration of ≈1×10^9^/ml. For conjugation of the anti-human and anti-mouse EpCAM antibody, maleimide-activated MBs were modified with anti-Fc fragment-specific IgG. Reactive sulfhydryl groups were introduced in anti-Fc IgG by reaction with Traut's reagent. The thiolated antibody was purified from excess Traut's reagent using a Zeba Spin Desalting Column. The number of thiol groups on the IgG molecules was determined with Ellman's reagent as described [22]. On average, each antibody had 1.5 thiol groups. Immediately after purification, the thiolated antibody was added to 2×10^8^ washed maleimide activated MBs. The conjugation was allowed to proceed for 1 hour at room temperature on a rotating plate set at a low speed. MBs were washed by centrifugation at low speed for three times. The final MB concentration was >10^8^ MB/ml. MBs were stored in PBS at 4°C prior to use. For magnetic bead coating with anti-EpCAM, 5 µm aminated magnetic beads in polystyrene matrix (Micromod, Rostock, Germany) were reacted with excess maleimide-PEG3400-SVA (Laysan Bio) for 1 hour, washed on a magnet, and reacted with the thiol-activated Fc-specific antibody and then anti-EpCAM antibody as described for MBs. Crosslinked iron oxide nanoparticles were prepared as described [23].

### 4. Quantification of IgG coupling and comparison between MBs and magnetic beads

The amount of IgG conjugated to the surface of MBs was quantified by SDS-PAGE under reducing conditions. MBs were dissolved in Laemmli sample buffer (Bio-Rad, Hercules, CA, USA) and destroyed in a water-bath sonicator; the amount equivalent to 3×10^7^ MBs was subsequently loaded on the gel (in a duplicate) and analyzed with SDS-PAGE and silver staining. For quantification of the band intensities, the IgG standard curve was prepared at the quantities of 4, 2, 0.667, 0.222, 0.074, and 0.025 µg IgG per lane. Detection of protein bands was performed with a Silver Quest staining kit (Invitrogen) according to the manufacturer's instructions. The silver-stained gels were scanned on a flatbed scanner and the intensity of the protein bands was quantified using ImageJ software. The density of 50 kDa bands (heavy IgG chain) was used for plotting the calibration curve and for sample concentration calculations. To compare the conjugation of rat anti-mouse EpCAM to MBs and beads, Alexa 488-labeled anti-rat IgG (Invitrogen) was used. After the labeling with the fluorescent antibody, MBs and beads were placed on a slide and multiple fluorescence images at 200×magnification were taken using the same exposure time. The background was subtracted and the integrated signal intensity of Alexa 488 on MBs and magnetic beads was determined using ImageJ freeware (Measure tool). The integrated intensity of each object was divided by pixel area of the same particle to obtain an average intensity/pixel value, which corresponds to the antibody density.

### 5. Cell culture

4T1 breast carcinoma cells were purchased from the American Type Culture Collection (ATCC; Manassas, VA, USA). GFP-positive 4T1 cells were kindly provided by Anticancer, Inc (San Diego, CA). ASPC-1 and A549 cells were from ATCC. Pancreatic cancer BxPC-3 cells were from the laboratory of Dr. Bouvet, UCSD [24]. GFP-PC3 prostate cancer cells were from the laboratory of Dr. Sugahara, Sanford-Burnham Medical Research Institute [25]. All cells were grown in RPMI 1640 supplemented with 10% heat-inactivated FBS and 100 mg/ml penicillin/streptomycin. All the cell lines were cultured at 37°C in a humidified incubator in the presence of 5% CO_2_.

### 6. Tumor cell binding

For MB and magnetic bead binding experiments, 1×10^4^ tumor cells were added to an eppendorf tube in 1 ml of complete cell medium, followed by the excess of anti-mouse EpCAM-MBs or anti-mouse EpCAM magnetic beads (particle/cell ratio ≈100/1). The cells and MBs/beads were mixed on a rotator at 10 rpm for various times. The binding efficiency was determined after taking an aliquot of the mixture and counting the percentage of MB/bead rosettes under microscope.

### 7. Feasibility of cell isolation with MBs

The minimal MB size, as well as the number of MBs to lift up a cell was calculated as follows. According to the Archimedes' law, the buoyancy force is proportional to the mass difference between an object (cell, MB) and the water that would occupy the same volume (excess mass, ***EM***). For the MB to be able to lift the cell upwards, the following condition should be fulfilled:



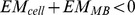



The excess mass of a cell (***EM_cel_***_l_) is:



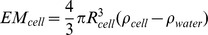



Similarly, the MB excess mass ***EM_MB_*** of radius ***R_MB_*** will be:



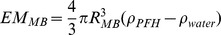



Where ρ_PFH_ is the density of perflorohexane gas (0.0106 g/ml), and ρ*_cell_* is the density of the cancer cell (1.08 g/ml [26]).

The number of antibodies to hold a tumor cell and a MB together was calculated from the tension force. When the MB-cell complex is not rising or floating, the tension ***T*** is the sum of the weight and the buoyancy force.



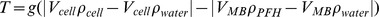



This equation does not take into account the drag forces. If the MB-cell complex is moving up, the drag on the cell will increase the tension, and the drag on the bubble will decrease the tension. For a 20 µm cell and a 9 µm MB at 300 g, the drag increases the tension by ∼13%.

### 8. Isolation of cells with MBs

For isolation experiments, anticoagulated (heparin) blood was obtained from healthy donors and metastatic cancer patients. Heparinized mouse blood was obtained from 6–15-week old BalB/C mice at the Moores UCSD Cancer Center vivarium. Blood was diluted 1∶5 with PBS and centrifuged at 2000 g for 20 minutes at room temperature, and plasma was carefully removed. The cells were then resuspended in PBS to bring the suspension to the initial blood volume. After this procedure, the concentration of plasma was decreased to less than 10%. Tumor cells were spiked into plasma-poor blood and MBs were added at 0.3-1×10^7^ MBs/ml (Dynabeads Epithelial Enrich protocol calls for 1×10^7^ beads/ml therefore magnetic beads were used at this concentration). The cells and MBs/beads were mixed on a rotator at 10 rpm for various times. Then, MBs were centrifuged at 100 g for 2 minutes, whereas beads were separated with external magnet.

For experiments with high concentration of tumor cells, MB layer after centrifugation was carefully harvested into an eppendorf tube containing 500 l of medium, and washed 2 times by centrifugation at 100 g. For magnetic beads, the slurry was washed 3 times and resuspended in 500 µl of medium. In some experiments, MBs were briefly (1 second) bath-sonicated to destroy MBs. Brief sonication does not destroy or damage the tumor cells. The total volume in the tube was measured, and the concentration of the GFP+ cells was determined by counting with hemocytometer.

To study the depletion of frequent tumor cells by flow cytometry, an aliquot of blood layer after separating the MB layer was collected, washed in PBS once and incubated in erythrocyte lysis buffer (Pierce) according to the manufacturer's instructions. The leukocytes and tumor cells were then resuspended in 1% BSA/PBS buffer and stained with Alexa Fluor 488-anti-mouse EpCAM antibody and PE-anti-mouse CD45 antibody according to manufacturer's instructions. The depletion of tumor cells was analyzed on a FACSCalibur instrument (BD Biosciences, San Jose, CA, USA) using FlowJo software.

For isolation and counting of rare spiked tumor cells, the top MB layer was carefully collected and transferred onto a slide. A Nikon E600 upright fluorescence microscope with SPOT RT color camera (4×magnification objective) was used to count the number of GFP-positive tumor cells on the slide. For detection of non-labeled tumor cells after isolation, MB layer was carefully transferred onto a nitrocellulose membrane in order to immobilize the isolated cells and to enable subsequent staining steps. MBs were destroyed by addition of ice-cold methanol, the membrane was blocked with mouse serum for 30 min and then stained for pan-cytokeratin (epithelial marker), Hoechst (nuclear marker) and optionally CD45 (leukocyte marker). For isolation of CTCs from cancer patients, 7.5 ml blood was drawn from metastatic cancer patients at the Moores Cancer Center, and the same procedure as described above was performed.

## Results

### 1. Preparation of EpCAM-targeted MBs

**Figure 1:**
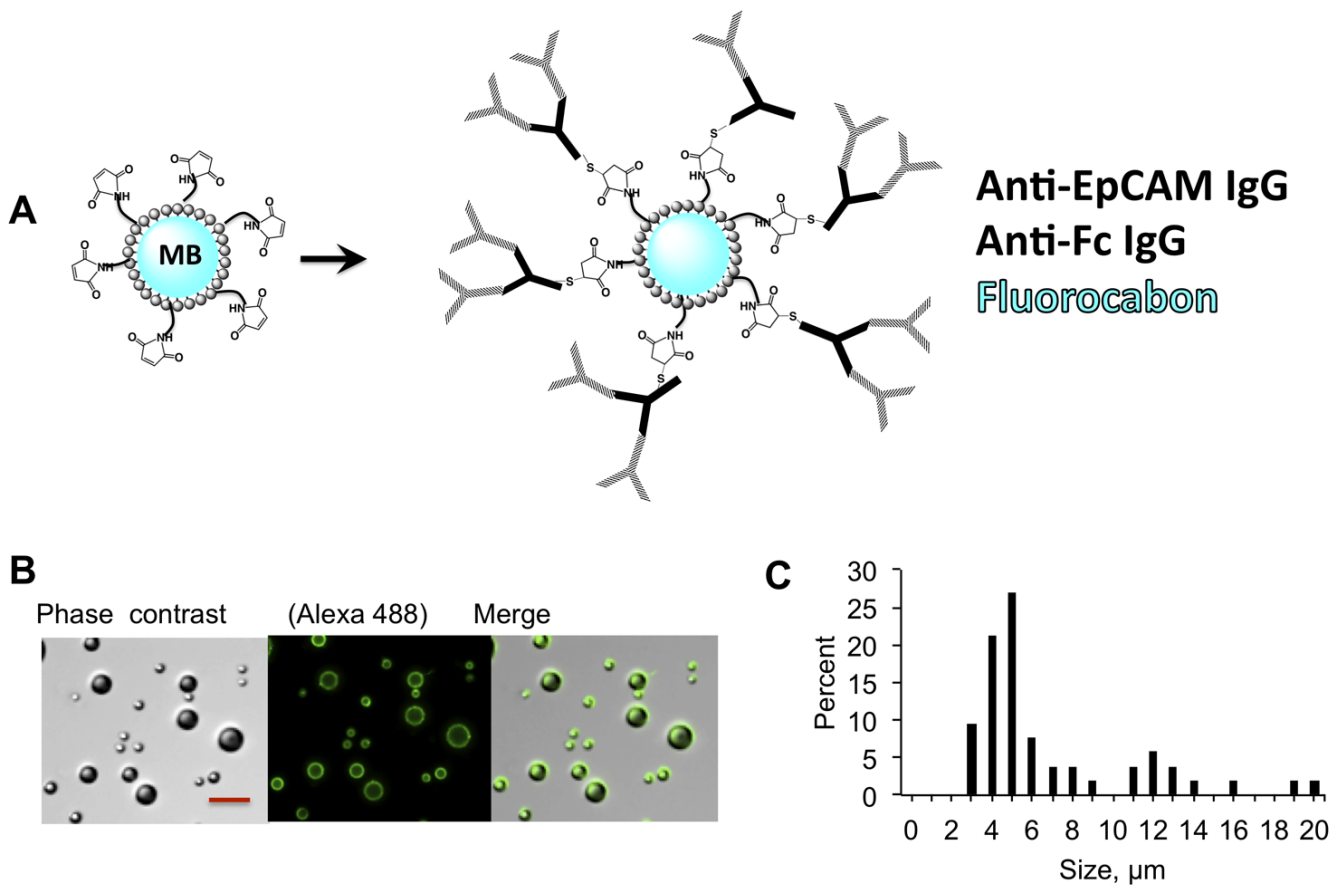
Synthesis of MBs for isolation. *A*, MBs were coated with anti-EpCAM antibodies in a two-step process as described in Methods; Based on the quantification (Supplement), on average 367,000 anti-Fc IgG molecules were coupled to the surface of each MB via Michael addition; *B*, anti-EpCAM was detected with Alexa 488-labeled secondary Ab. Size bar, 20 µm; ***C***, Size distribution of IgG coated MBs as determined from microscopy images.

We prepared MBs modified with anti-EpCAM IgG as shown in **Figure 1A**. The preparation of targeted MBs involved a two-step conjugation. First, we conjugated the anti-Fc antibody to MBs via maleimide chemistry, and then added the anti-EpCAM antibody. After the conjugation and washing steps steps, MBs were larger than 2 µm, with 60% of MBs sized between 3 and 8 µm (**Fig. 1B**), and the median size of 5 µm. MBs prepared by the emulsification method usually result in a broad size distribution [27]; microfluidic manufacturing methods could be utilized in the future to control MB size [28]. As determined by Western blotting (**Fig. S1**), on average each MB had 3.7×10^5^ PEG-maleimide-coupled anti-Fc IgG, which theoretically should correspond to 7.4×10^5^ anti-EpCAM IgG.

### 2. Binding of MBs to tumor cells

**Figure 2:**
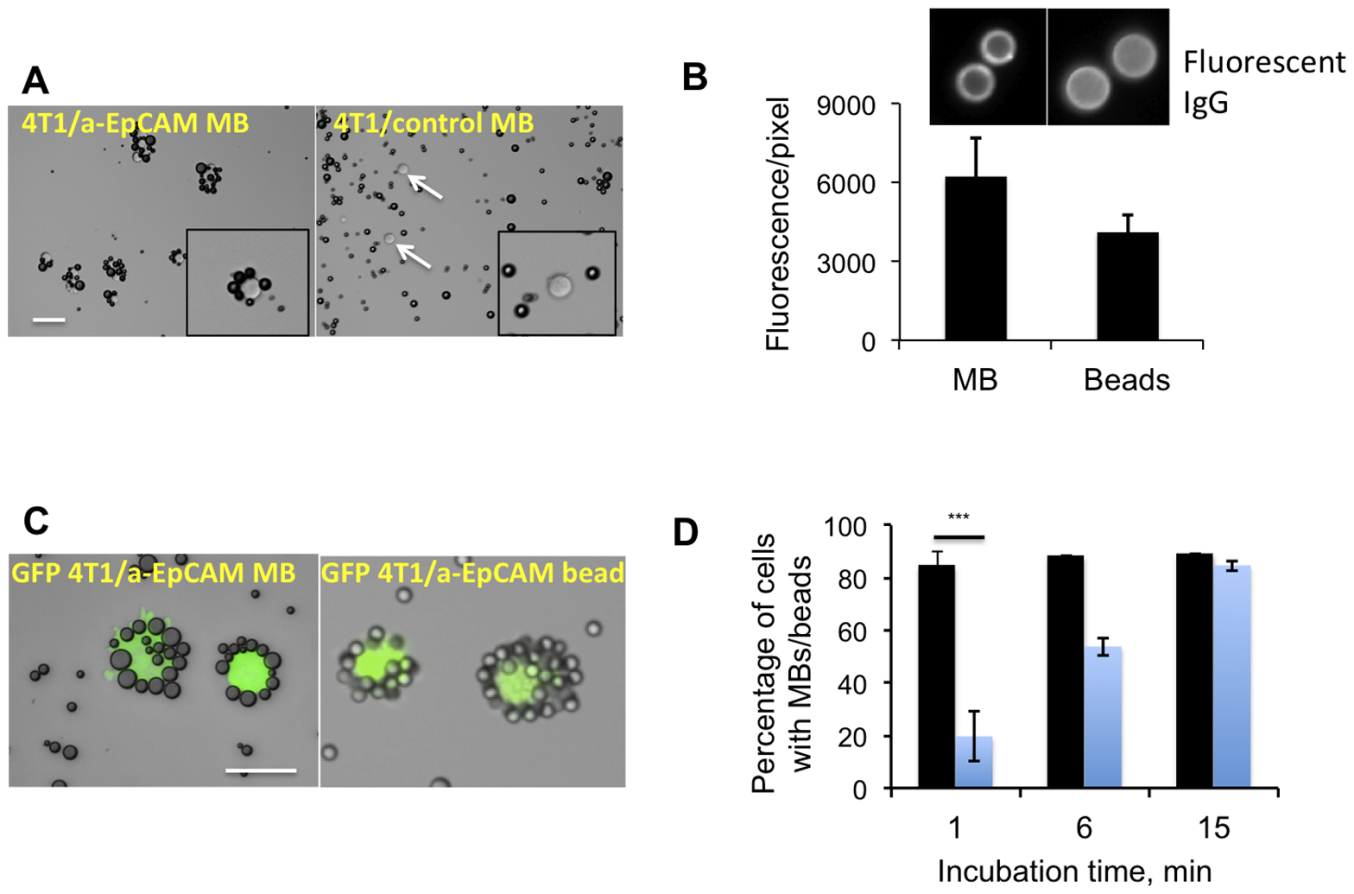
Binding of MBs to cultured tumor cells. *A*, Anti-EpCAM MBs and control MBs were added at 100∶1 ratio to a suspension of 100,000 4T1 mouse breast carcinoma cells in 1 ml cell medium and mixed for 15 min. Targeted MBs formed rosettes around the cells, while control MBs did not show any binding. Size bar, 50 µm for both images; *B*, Magnetic beads (5 µm diameter) were decorated with the anti-EpCAM antibody according to the strategy described in Fig. 1. Anti-EpCAM IgG was detected on MBs and beads using a secondary fluorescent antibody; the coating was comparable for MBs and beads; *C*, MBs and beads were mixed with GFP-4T1 cells in 1 ml medium (100∶1 ratio). The characteristic rosettes between MBs and beads were observed after 15 min; ***D***, Binding efficiency of MBs and magnetic beads was determined at different time points. Blue bars, percentage of cells coated with beads; black bars, percentage of cells coated with MBs. After 1 min, anti-EpCAM MBs bound to 4T1 cells more efficiently than anti-EpCAM magnetic beads (t-test, P = 0.0001, n = 3).

In order to test the binding of MBs to tumor cells in suspension, we first used mouse breast carcinoma 4T1 cells. These cells are of epithelial origin and we verified that over 95% of the cells express EpCAM (albeit the expression was heterogeneous, **Fig. S2**). MBs were added to cells in 1 ml medium at 100∶1 ratio. Following 1 hour of gentle mixing, the cells formed ‘rosettes’ with anti-EpCAM MBs (**Fig. 2A**). Anti-EpCAM MBs attached to over 80% of cells while non-targeted MBs did not show any appreciable binding (**Fig. 2A**). In addition to mouse 4T1 cells, we tested the binding to other epithelial cell lines, as summarized in **Table 1**. Anti-human EpCAM MBs efficiently bound to prostate cancer GFP-PC3 cells, lung adenocarcinoma A549 cells, and pancreatic adenocarcinoma BxPC-3 and ASPC-1 cells but did not appreciably bind to non-epithelial lymphoma JeKo-1 cells. In order to compare side-by-side the binding efficiency of MBs with immunomagnetic beads, a current gold standard for the cell isolation, we prepared 5 µm diameter anti-mouse EpCAM magnetic beads using the same two-antibody approach (see Methods) and the same PEG linker (PEG3400) as for MBs. The conjugation resulted in comparable amounts of anti-EpCAM IgG on MBs and beads (**Fig. 2B** **, Fig. S3**). MBs or beads were added to GFP-tagged 4T1 cells at 100∶1 ratio and mixed. Following 15 min incubation, 89.2% of tumor cells became coated with MBs and 84.7% of cells coated with beads (**Fig. 2C-D**). Notably, MBs formed rosettes with 82% of cells within 1 min incubation, suggesting that MB–cell binding is very efficient and fast (**Fig. 2D**). The fast binding kinetics is demonstrated in **Movie S1**. However, there were always 10-15% of cells that were not coated by MBs or beads. It is likely that these cells express lower numbers of EpCAM molecules (**Fig. S2**) and therefore more difficult to bind.

**Table 1:** Tumor cell lines tested for anti-EpCAM MB binding.

Name	Description	MB binding efficiency
4T1	Mouse breast carcinoma, epithelial	>85%
GFP-4T1	Mouse breast carcinoma, epithelial	>85%
BxPC3	Human pancreatic adenocarcinoma, epithelial	>90%
A549	Human lung adenocarcinoma, epithelial	>90%
ASPC-1	Human pancreatic adenocarcinoma, epithelial	>90%
GFP-PC-3	Human prostate cancer, epithelial	>90%
JeKo-1	Lymphoma, non-epithelial	<2%

### 3. Theoretical aspects of tumor cell isolation

**Figure 3:**
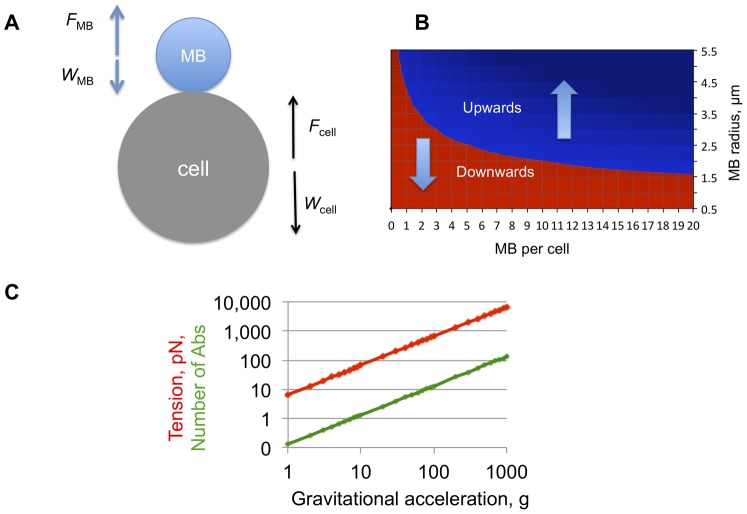
Theoretical feasibility of cell flotation after MB attachment. The number of MBs to pull up a cell and the force acting on the MB-cell attachment was calculated based on the buoyancy law as described in Methods. *A*, In a non-viscous aqueous medium, cells and MBs experience the buoyant force (F) and the gravitation force or weight (W). Tension (force acting to disrupt MB–cell attachment) is the sum of F and W. This scheme does not take into account the forces acting in blood, such as drag (viscous resistance) and cell-cell interactions; *B*, Phase diagram showing the balance of gravity and buoyant forces at different MB sizes and MB/cell ratios. Cell diameter of 20 µm was used for the calculations. In the red zone, the weight of MB-cell complexes prevails and the complexes sediment; in the blue zone the buoyancy takes over and the complexes float. Gravitational acceleration does not affect the direction of the MB-cell complexes but only increases the speed of movement and tension force; ***C***, Tension between a single 9 µm-diameter MBs and a 20 µm cancer cell as a function of g-force. Red line is the tension force; green line is the calculated number of antibodies required for holding a MB and a cell together (assuming that the force to pull out a phospholipid from the membrane is equal to 50 pN [29], [30], [31]).

We calculated the minimum size and number of MBs that need to be attached to a tumor cell to enable the flotation. We performed the calculation based on the balance of buoyancy and weight forces acting on MBs and cells (**Fig. 3A** and Methods). According to the Archimedes law, the flotation of cancer cells in aqueous medium critically depends on the MB size and the number of MBs per cell. For a 20 µm diameter cell, a single 9 µm diameter MB can pull the cell up (**Fig. 3B**). Since the median size of our MBs was 5 µm, 5 MBs should be sufficient to lift up a 20 µm cell (**Fig. 3B**). Our binding data (**Fig. 2**) showed that on average there were more than 5 MBs per cell, suggesting that the isolation of cancer cells is practically feasible.

When targeted MBs become attached to the cell surface, buoyancy and gravity forces create a tension on the MB–cell attachment (**Fig. 3A**). The tension forces could be calculated and the number of antibodies to withstand the tension force could be estimated. For a single 9 µm MB attached and a 20 µm cell, the calculated tension force at 1 g (no centrifugation) is 6.7 pN (**Fig. 3C**). The force needed to disrupt one antibody-receptor bond is between 50-80 pN [29], [30]. The force needed to pull a lipid molecule out of a lipid bilayer is similar [31]. That means a single Ab is sufficient to hold the MB–cell complex together when no centrifugation force is applied. At 100 g, about 13 antibodies would be needed to hold the MB and the cell. The chance of breaking the MB-cell connection increases linearly (if ignoring drag forces) with centrifugation speed. At 1000 g, over 130 antibodies would be required to hold the MB–cell complex together (**Fig. 1C**). The actual number of EpCAM molecules at the MB–cell contact (0.2–0.5 µm^2^) is well below 100 [9]. Although the tension forces decrease significantly in case multiple MBs are attached per cell, we limited the centrifugation to 100 g in the actual isolation experiments.

### 4. Isolation of frequent spiked tumor cells with MBs and beads

**Figure 4:**
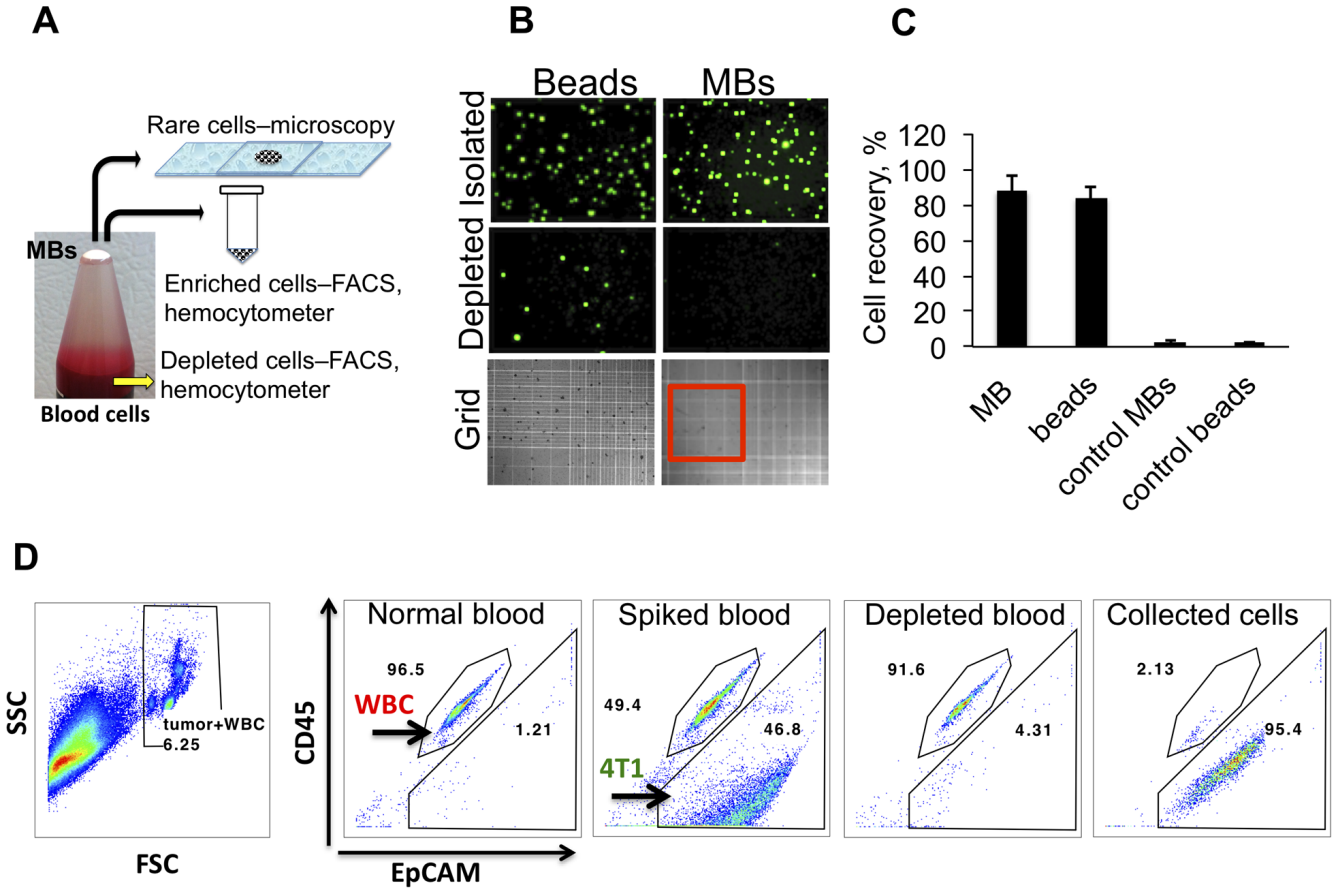
Isolation of tumor cells with MBs and magnetic beads. Tumor cells were spiked into 1 ml of plasma-depleted mouse blood (see Methods). Mouse EpCAM-targeted MBs and beads were added at 1×10^7^ MB/ml. Following gentle mixing for 15 minutes, MB layer was separated from blood cells by centrifugation at 100 g for 2 minutes. Magnetic beads were separated with a neodymium magnet. ***A***, Collection of the floating MB layer on top depends on the downstream analysis (see Methods); ***B***, After the isolation of MBs and beads, the cells were placed on a hemocytometer grid (MBs were destroyed for 1 second with gentle sonication) and the GFP-positive cells were counted at low magnification (40×). MBs and beads showed similar numbers of GFP+ cells in the isolated (enriched) fraction and near absence of GFP+ cells in the blood cell (depleted) fractions. For size reference, red outline shows the major 5×5 square of the hemocytometer; ***C***, Quantification of isolation efficiency of GFP-4T1 cells. ***D***, Quantification of tumor cell depletion from blood cells with flow cytometry. One ml of blood cells was spiked with non-labeled 4T1 cells at 1×10^6^ cells/ml. Remaining blood cell fraction and the isolated MB fraction were analyzed for tumor cells after CD45 (leukocyte) and EpCAM staining (as described in Methods). Red blood cells were lysed and gated out (left image, FSC/SSC plot). MBs efficiently depleted 4T1 cells, and enriched them with 95.4% purity. A representative experiment out of two is shown.

To perform actual isolation of tumor cells from complex cell suspensions, we spiked various amounts of tumor cells into plasma-depleted (<10% plasma) blood. We used plasma-depleted blood (hereafter ‘blood cells’) because MB stability is somewhat decreased in whole blood (not shown), possibly due to gas mixing and exchange [32]. Two sets of experiments were performed. In order to reliably quantify the isolation efficiency with hemocytometer or flow cytometry, the first set of experiments was done with high concentration of spiked tumor cells (100,000-1,000,000 cells/ml). High number of cells lowers the uncertainty and experimental error associated with spiking rare cells. The second set of experiments was performed with rare tumor cells (13-24 cells/ml). **Figure 4A** demonstrates the workflow of our experiments. Following isolation (the full procedure for MB collection will be published elsewhere), MB layer was collected either into an eppendorf tube for FACS and hemocytometer, or directly on a slide for counting of rare cells (**Fig. 4A**).

For testing the isolation efficiency, 100,000 cells GFP-4T1 cells were added to 1 ml blood cells followed by EpCAM MBs or magnetic beads. Following isolation, the cells were counted with hemocytometer (**Figure 4B** **).** EpCAM-targeted MBs and beads isolated GFP-4T1 cells from blood cells with high efficiency (88±8.5% for MBs and 84±6% for beads). Control IgG MBs and beads isolated 2.5±1.5 and 2.1±0.8 tumor cells, respectively (**Fig. 4C**). To quantify the depletion and enrichment of tumor cells with flow cytometry, 1×10^6^ GFP-4T1 cells were spiked into 1 ml mouse blood cells and isolated with anti-EpCAM MBs. According to **Fig. 4D**, EpCAM+ cells constituted 46.8% of non-RBC blood cells before, but only 4.31% after the addition and separation of anti-EpCAM MBs (corresponding to about 90% depletion efficiency). In the MB-enriched sample, tumor cells comprised 95.4% of all cells (**Fig. 4D** **)**, corresponding to a 47-fold enrichment.

MBs and beads carried over some white blood cells (WBCs) in these experiments. The number of WBCs was variable and was dependent on experimental conditions. WBC carryover was consistently higher for micron-sized magnetic beads than for MBs or nano-sized crosslinked iron oxides (**Fig. S4A**). Interestingly, in the MB layer some of the WBCs were passively entrapped and not bound to the outer membrane of MBs (**Fig. S4B**), suggesting that efficient washing steps could further decrease the non-specific carryover.

### 5. Isolation of rare spiked cells and patients' tumor cells with MBs

**Figure 5:**
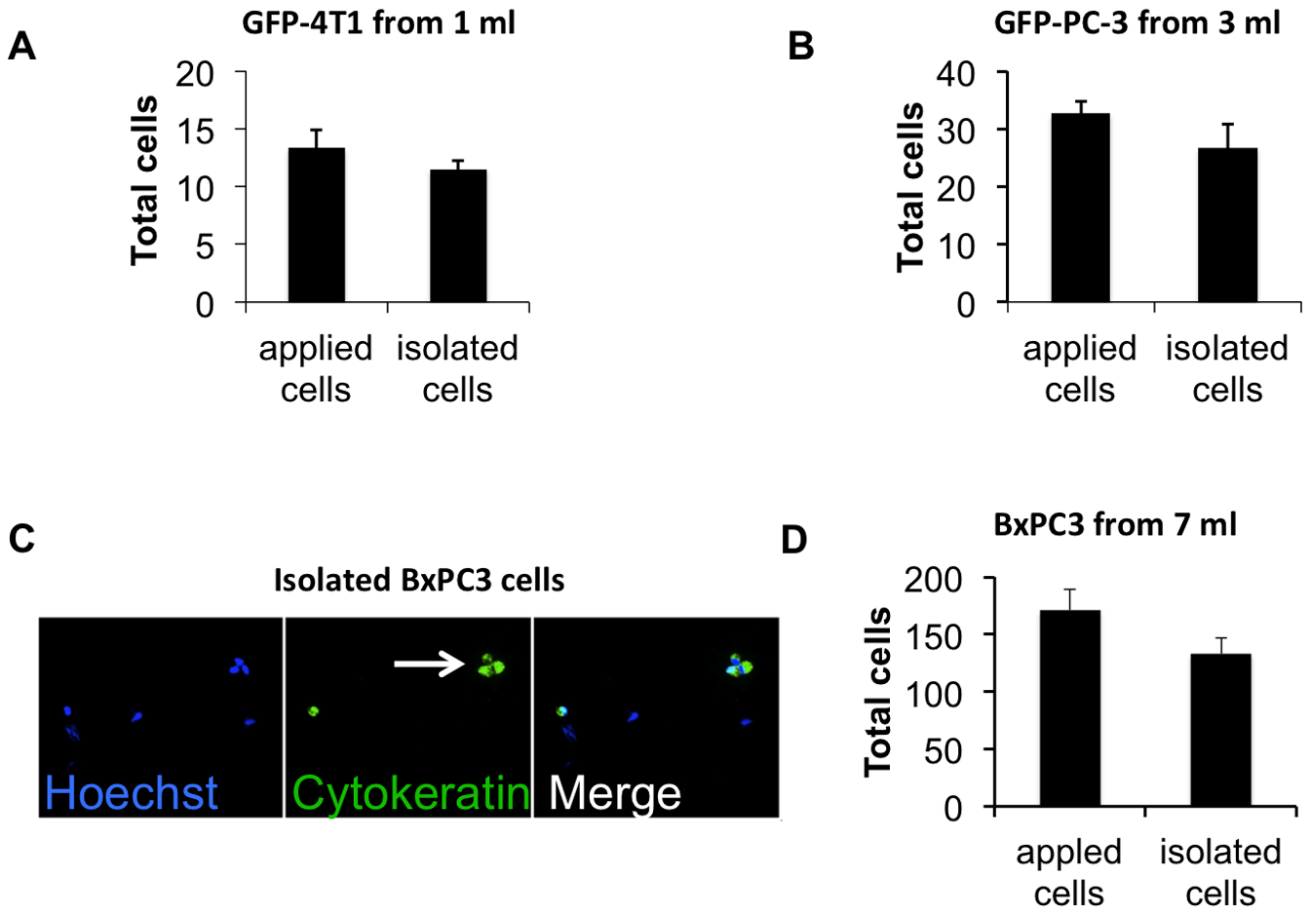
Isolation of rare cells with MBs. Rare tumor cells were added to plasma-depleted blood, isolated with MBs as described in Fig. 4A and counted on a slide. In order to avoid spiking and counting errors, the same number of tumor cells that was added to blood cells prior to isolation (typically in 5–10 µl volume) was placed on a slide and counted in parallel with the isolated sample; ***A***, Mouse breast cancer GFP-4T1 cells were added to 1 ml blood and isolated with anti-mouse EpCAM MBs (n = 3). ***B***, Prostate cancer GFP-PC-3 cells were added to 3 ml of plasma-depleted blood and isolated with anti-human EpCAM MBs (n = 3); ***C***, Pancreatic cancer BxPC3 cells were added to 7 ml plasma-depleted blood and isolated with anti-human EpCAM MBs. Unlike the experiments with GFP-tagged cells, the isolated cells were stained with pan-cytokeratin antibody. A representative microscopic field (20×objective) shows the MB-isolated BxPC3 cells positive for CK (green). Hoechst-positive, CK-negative cells, which are presumably carryover leukocytes, are also visible in the field. Arrow points to a tumor cell cluster; ***D***, There was a 77% efficiency of isolation of BxPC3 cells from 7 ml (n = 3).

CTCs are present in blood of metastatic cancer patients at extremely low concentrations of a few cells per milliliter [11]. In order to determine the isolation efficiency of rare cells, we spiked plasma-depleted blood at a concentration of 13-24 tumor cells per ml and recovered them with anti-EpCAM MBs. We used the approach described in Fig. 4A to isolate and count the MB-attached cells. In order to decrease the spiking error, we placed on a slide the same number of cells as used for spiking and counted in parallel with the isolated cells. When we added 13 GFP-4T1 cells into 1 ml blood cells and isolated with anti-mouse EpCAM MBs, (**Fig**. **5A** **)**, MBs recovered 86.4% of the cells (n = 3). When human prostate GFP-PC3 cells were spiked into 3 ml blood cells (11 cells/ml) and isolated with anti-human EpCAM MBs, 81.9% of the cells (n = 3) were recovered (**Fig. 5B**). In order to mimic the protocol for isolation of CTCs from human patients (7.5 ml blood volume is the current standard for CTC isolation using Veridex CellSearch kit [11]), we spiked 171 non-labeled human pancreatic adenocarcinoma BxPC3 cells into 7 ml of plasma depleted blood (24 cells/ml) and isolated them with 3×10^7^ MBs. The difference from the 1 ml and 3 ml experiments was that the tumor cells post-isolation were stained for pan-cytokeratin (epithelial marker) for identification. The tumor cells were CK-positive/Hoechst-positive, whereas contaminating leukocytes were CK- negative (**Fig. 5C**). Interestingly, MBs also isolated BxPC-3 cell clusters (**Fig. 5C**, arrow). According to **Fig. 5D**, MBs isolated 77.8% of the applied cells (n = 3). No CK-positive cells were isolated from samples that were not spiked with tumor cells (not shown).

**Figure 6:**
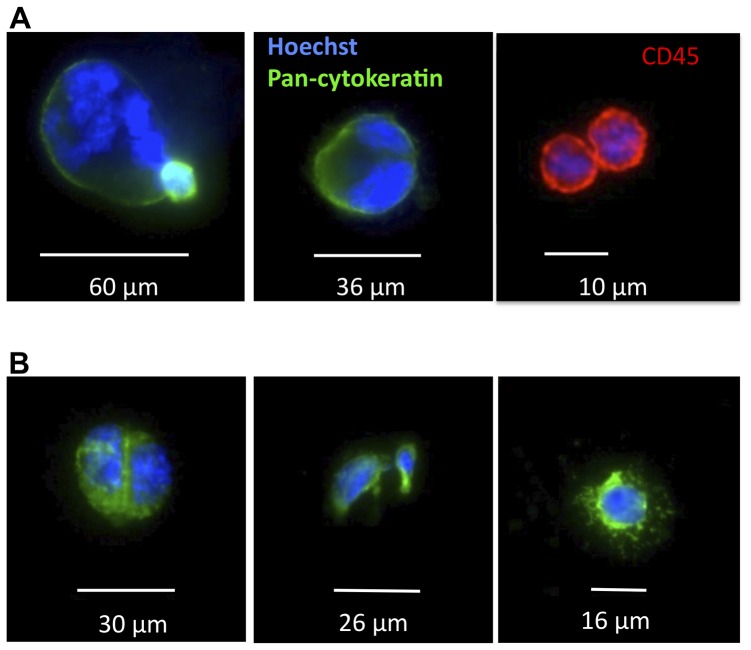
Isolation of CTCs from blood of metastatic cancer patients. ***A***, Case 1 was invasive, moderately to poorly differentiated esophageal adenocarcinoma with brain metastases; ***B***, Case 2 was metastatic clear cell carcinoma with brain metastases. In both cases, CK+/CD45- cells, which are presumably CTCs were identified (also **Fig. S4**). Some cells present mitotic figures and multinuclear morphology as described before [34]. CK-/CD45+ cells (presumably leukocytes) are also shown in a separate field (red).

As a preliminary study, the ability of targeted MBs to detect CTCs from 7 ml blood of metastatic cancer patients was tested. Case 1 was invasive, moderately to poorly differentiated esophageal adenocarcinoma with brain metastases. Case 2 was metastatic clear cell carcinoma with brain metastases. Based on the established CTC isolation methods [10], [11], CK+/CD45+ nucleated cells were considered tumor cells, whereas CK-/CD45+ nucleated cells were categorized as leukocytes. As shown in **Figure 6A-B** and in **Figs. S5A-B**, in both cases anti-EpCAM MBs isolated CK-positive, CD45-negative cells (21 and 8 cells, respectively).

## Discussion

CTCs are quickly becoming a valuable diagnostic and prognostic marker for individualized medicine [33]. Besides counting CTCs numbers of as a prognostic marker, genetic profiling and expression analysis of CTCs are a promising approach for cancer prognosis and drug screening [33]. CTC CTCs could be used for ‘blood biopsy’, i.e., exfoliated CTC are used instead of tissue biopsy for diagnosis and drug testing. Since CTCs are extremely rare and non-homogenous, sample preparation is a crucial parameter for downstream analytical techniques.

Here, we demonstrate that epithelial tumor cells could be isolated from plasma-depleted blood using buoyant microbubbles targeted to epithelial marker on the surface of tumor cells. To our best knowledge, such a study has not been reported in literature. Using EpCAM targeted MBs we achieved 88% isolation efficiency of tumor cells spiked at high concentration (100,000 cells/ml) in 1 ml of plasma-depleted blood, and 77% isolation efficiency of rare cells (23 cells/ml) spiked in 7 ml of plasma-depleted blood. In addition to the spiking experiments, we were able to detect cytokeratin-positive cells in blood of metastatic cancer patients, including dividing and a multinuclear cells, similar to what was reported for metastatic cancers [34]. It must be stressed that currently accepted approach of identification of circulating tumor cells relies on the presence of pan-cytokeratin epithelial marker that does not identify CTCs per se, and more specific markers are needed to positively identify tumor cells [35]. Based on the large size of some of the isolated cells, high nucleus/cytoplasm ratio, irregular shape of the nuclei, and appearance of mitotic figures we suggest that MB-isolated cells are indeed tumor cells.

Sample preparation is one of the most critical aspects of biospecimen analysis. Several important properties of MBs could be very useful for CTC sample preparation, including speed, potential scalability and simplicity. Thus, MB-cell binding takes place faster than comparable immunomagnetic beads (within a few minutes) as evidenced in **Fig. 2D** and **Movie S1**. For example, speed of processing can improve the quality of tissue expression analysis of CTCs. [36] Another important property of perfluorocarbon MBs is that they are easy to eliminate in the final sample (mild sonication or negative pressure), compared with magnetic beads or microfluidic chip where the cells need to be recovered with a proteolytic enzyme [17]. Non-specific contamination with leukocytes is another serious problem for immunomagnetic CTC isolation [37], [38]. Reducing a non-specific cell contamination is critical for single genome analysis that usually requires >1% purity of the target gene [38]. MBs resulted in some carryover of leukocytes, which was less than with 5 µm magnetic beads and similar with CLIO nanoparticles used in this study (**Fig. S4**). Since there are many factors that affect isolation purity, including surface properties (polystyrene vs. phospholipid vs. hydrogel), cell concentration and washing, more extensive comparison of purity of isolation needs to be performed before drawing any conclusions regarding purity of this or that method. Additional optimization of incubation/washing/collection steps as well as improved MB formulation could further increase the level of sample purity in order to enable sensitive and specific analysis of the CTC genome. So far, the main limitation of the method is MB instability in whole blood, necessitating washing steps prior to application of MBs. Some of the instability of MBs in whole blood is possibly due to gas mixing and exchange [32]. With correct formulation and gas composition the stability of MBs could be significantly improved [39].

We are confident that the technical challenges could be solved and MBs could evolve into an attractive CTC isolation approach for personalized medicine.

## Supporting Information

Figure S1 **Quantification of IgG on MBs.** ***A***, MBs were destroyed in a water-bath sonicator, the amount equivalent to 3×10^7^ MBs was loaded on the gel (in a duplicate) and analyzed with SDS-PAGE and silver staining. For quantification of the band intensities, the IgG standard curve was prepared at the quantities of 4, 2, 0.667, 0.222, 0.074 and 0.025 µg IgG per lane. ***B***, Standard curve was generated by measuring integrated band intensity with ImageJ software. Based on the quantification, on average 367,000 antibody molecules were coupled to the surface of each MB via Michael addition.
(TIFF)

Figure S2 **EpCAM expression on 4T1 cells.** Analysis of EpCAM levels was performed by FACS analysis. Per sample, 1.0×10^6^ cells were stained with Alexa-488 conjugated mouse anti-human EpCAM antibody for 30 minutes at 4°C. Cells were then washed three times with FACS buffer. All the samples were analyzed on a FACSCalibur instrument (BD Biosciences, San Jose, CA, USA). The acquisition was set to 50,000 events. The cell population was gated on a FSC/SSC plot, and mean fluorescence intensity and percentage of FL-2 positive cells was determined using FlowJo software.
(TIFF)

Figure S3 **Anti-EpCAM coating of MBs and beads.** This is a representative non-cropped image from **Fig. 2B** of fluorescent antibody-stained MBs and beads for the comparison of anti-EpCAM coating.
(TIFF)

Figure S4 **Non-specific carryover of white blood cells (WBCs) with MB fraction.** IgG MBs (1×10^7^), IgG magnetic beads (1×10^7^) and IgG CLIO were added to 7 ml of plasma-depleted human blood and incubated for 15 minutes at RT. MBs were separated as described in Fig. 4; magnetic beads were separated with a magnet, CLIO were separated with Miltenyi MIDI column. Nucleated cells (white blood cells) were stained with Hoechst nuclear dye and counted with a hemocytometer. ***A***, Amounts of WBCs after isolation. WBCs are present in all fractions, but magnetic bead fraction consistently contained significantly more WBCs that MBs and CLIO. Size bar, 100 µm; ***B***, The image shows that some WBCs are not attached to MBs.
(TIFF)

Figure S5 Images of CK+/CS45- cells the MB layer after isolation from metastatic cancer samples. A and B correspond to Fig. 6A-B.
(TIFF)

Movie S1 **Isolation of tumor cells from PBS.** Formalin-fixed, DiI-labeled 4T1 cells (approximately 1×10^6^) were added to 3 ml PBS, followed by 3×10^7^ EpCAM-targeted or control MBs. The cells were mixed and allowed to separate. The targeted MB bound and separated cells within 7 min.
(MOV)
