# Supplementary figures and images for: Isolation of Rare Tumor Cells from Blood Cells with Buoyant Immuno-Microbubbles
Source: PLoS One. 2013 Mar 13;8(3):e58017. doi: 10.1371/journal.pone.0058017 (PMC3596333; doi:10.1371/journal.pone.0058017)

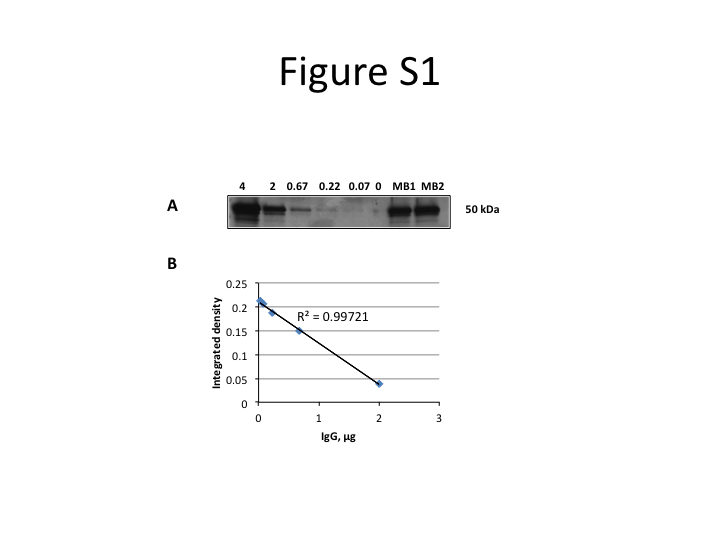

Supplement: Figure S1 — Quantification of IgG on MBs. A, MBs were destroyed in a water-bath sonicator, the amount equivalent to 3×10^7^ MBs was loaded on the gel (in a duplicate) and analyzed with SDS-PAGE and silver staining. For quantification of the band intensities, the IgG standard curve was prepared at the quantities of 4, 2, 0.667, 0.222, 0.074 and 0.025 µg IgG per lane. B, Standard curve was generated by measuring integrated band intensity with ImageJ software. Based on the quantification, on average 367,000 antibody molecules were coupled to the surface of each MB via Michael addition. (TIFF) [file pone.0058017.s001.tiff]

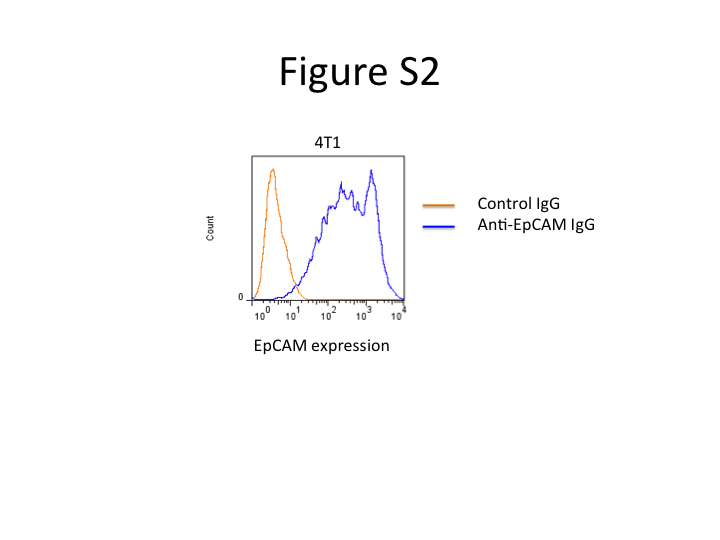

Supplement: Figure S2 — EpCAM expression on 4T1 cells. Analysis of EpCAM levels was performed by FACS analysis. Per sample, 1.0×10^6^ cells were stained with Alexa-488 conjugated mouse anti-human EpCAM antibody for 30 minutes at 4°C. Cells were then washed three times with FACS buffer. All the samples were analyzed on a FACSCalibur instrument (BD Biosciences, San Jose, CA, USA). The acquisition was set to 50,000 events. The cell population was gated on a FSC/SSC plot, and mean fluorescence intensity and percentage of FL-2 positive cells was determined using FlowJo software. (TIFF) [file pone.0058017.s002.tiff]

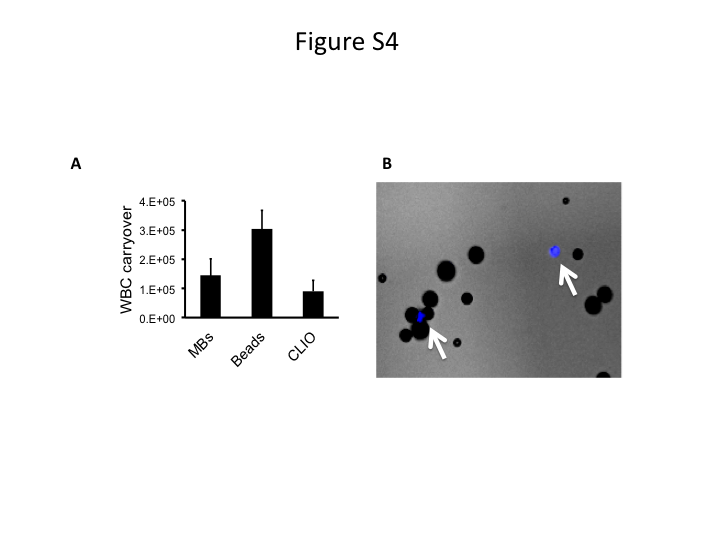

Supplement: Figure S4 — Non-specific carryover of white blood cells (WBCs) with MB fraction. IgG MBs (1×10^7^), IgG magnetic beads (1×10^7^) and IgG CLIO were added to 7 ml of plasma-depleted human blood and incubated for 15 minutes at RT. MBs were separated as described in Fig. 4; magnetic beads were separated with a magnet, CLIO were separated with Miltenyi MIDI column. Nucleated cells (white blood cells) were stained with Hoechst nuclear dye and counted with a hemocytometer. A, Amounts of WBCs after isolation. WBCs are present in all fractions, but magnetic bead fraction consistently contained significantly more WBCs that MBs and CLIO. Size bar, 100 µm; B, The image shows that some WBCs are not attached to MBs. (TIFF) [file pone.0058017.s004.tiff]

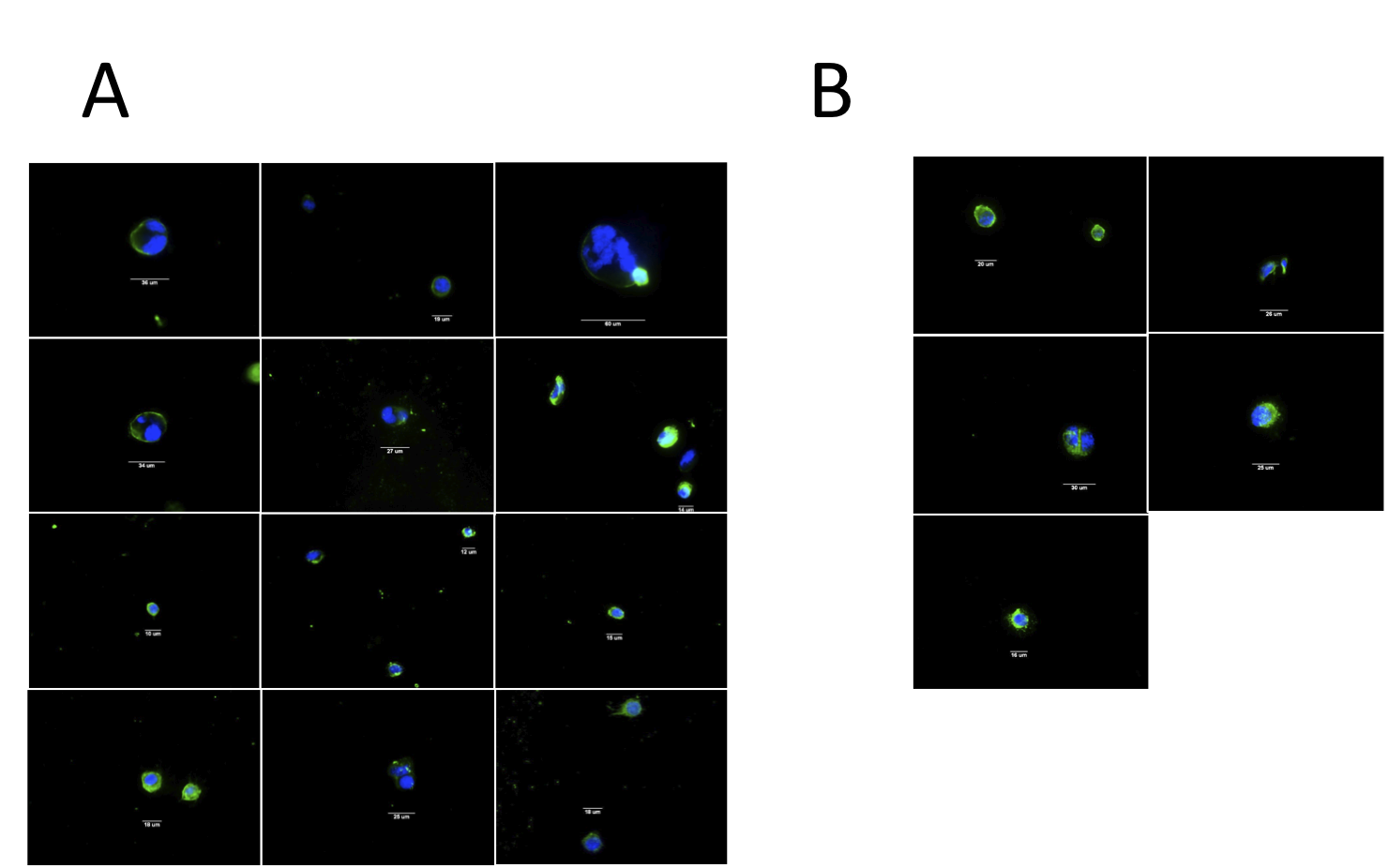

Supplement: Figure S5 — Images of CK+/CS45- cells the MB layer after isolation from metastatic cancer samples. A and B correspond to Fig. 6A-B. (TIFF) [file pone.0058017.s005.tiff]
